# The diagnostic value of endobronchial ultrasound-guided transbronchial needle aspiration for bronchial anthracosis/anthracofibrosis with mediastinal enlarged lymph node

**DOI:** 10.3389/fmed.2025.1603922

**Published:** 2025-08-04

**Authors:** Xiao Li, Jinbing Pan, Ying Ren, Pei Wang, Guannan Sun

**Affiliations:** ^1^Department of Respiration, Henan Provincial People's Hospital, Zhengzhou, China; ^2^Department of Pathology, Henan Provincial People's Hospital, Zhengzhou, China

**Keywords:** bronchoscope, bronchial anthracosis, endobronchial ultrasound-guided transbronchial needle aspiration, anthracosis pigmentation, bronchial anthracofibrosis

## Abstract

**Objective:**

To explore the value of endobronchial ultrasound-guided transbronchial needle aspiration (EBUS-TBNA) in patients of bronchial anthracosis (BAC)/bronchial anthracofibrosis (BAF) with enlarged mediastinal lymph nodes.

**Methods:**

A retrospective analysis was conducted on 5,589 bronchoscopies performed between January and December 2023. Among them, 62 patients were diagnosed with BAC, including 30 cases of BAF. Patients were categorized into BAF and non-BAF groups. Clinical data, laboratory findings, high-resolution computed tomography (HRCT) results, bronchoscopic features, and EBUS-TBNA outcomes were analyzed and compared between the two groups.

**Results:**

The overall incidence rates of BAC and BAF were 1.11 and 0.54%, respectively, with BAF accounting for 48.4% of all BAC cases. Compared to the non-BAF group, the BAF group exhibited higher frequencies of bronchial stenosis, mediastinal lymphadenopathy, and lymph node calcifications on HRCT. EBUS-TBNA cytology predominantly revealed lymphocytic infiltration and pigment-laden macrophages, with some findings of granulomatous inflammation and malignant cells. There was no significant difference in the overall diagnostic yield of EBUS-TBNA between the two groups.

**Conclusion:**

EBUS-TBNA is a highly valuable diagnostic tool for differentiating BAC/BAF with mediastinal lymphadenopathy. It provides crucial pathological insights, especially in cases potentially involving malignancy or granulomatous disease.

## 1 Introduction

Bronchial anthracosis (BAC) results from the inhalation of exogenous dust, causing visible pigmentation of the bronchial mucosa or submucosa during bronchoscopy. When accompanied by bronchial structural changes, such as thickened walls, narrowed/ tortuous lumens, or even occlusion—the condition is termed bronchial anthracofibrosis (BAF) (1). Some patients present with enlarged mediastinal lymph nodes (LNs), requiring careful differential diagnosis. Advanced imaging techniques like Positron Emission Tomography-Computed Tomography (PET-CT) can suggest malignancy when nodes show high metabolic activity but cannot exclude benign conditions (2). Endobronchial ultrasound-guided transbronchial needle aspiration (EBUS-TBNA) has emerged as a pivotal diagnostic tool for evaluating enlarged mediastinal lymph nodes(LNs). By clearly delineating vascular structures, EBUS-TBNA minimizes the risk of vascular injury while ensuring precise lesion localization. Furthermore, integration of color Doppler ultrasound improves the accuracy of both procedural navigation and diagnostic outcomes (3). There remains a paucity of clinical research exploring the role of EBUS-TBNA in diagnosing BAC and BAF associated with enlarged mediastinal LNs (4). To address this gap, we conducted a retrospective analysis of patients who underwent bronchoscopy at our institution in 2023. Through detailed examination of BAC and BAF cases, we elucidate their clinical characteristics. In select patients, EBUS-TBNA was used to obtain pathological samples from mediastinal LNs. Additionally, bronchoalveolar lavage fluid (BALF) analysis was performed to further characterize bronchial anthracosis/anthracofibrosis in the setting of enlarged mediastinal LN lesions. These findings aim to provide valuable insights for clinical diagnosis and treatment strategies.

## 2 Participants and methods

### 2.1 Participants

From January 2023 to December 2023, a total of 5,589 bronchoscopy examinations were conducted in the bronchoscopy operating room of Henan Provincial People’s Hospital. 62 patients exhibited BAC, as confirmed by diagnostic criteria. Of these, 30 cases presented with airway stenosis that met the specific diagnostic criteria for BAF (1). The patients were subsequently divided into two groups: the BAF group and the non-BAF group. This approved by the Ethics Committee of Henan Provincial People’s Hospital, with a waiver of informed consent granted (1). All patients included in the study were followed up for one year to establish their final clinical diagnoses.

### 2.2 Criteria

#### 2.2.1 Diagnostic criteria for BAC

Pigmentation was observed on the local mucosa of the tracheobronchial tree during bronchoscopy (1).

#### 2.2.2 Diagnostic criteria for BAF

Under bronchoscopy, varying shapes and extents of pigmentation spots were observed in the submucosal layer of the large airways, including the trachea, main bronchi, and lobar segmental bronchi. There was evidence of localized or diffuse stenosis or occlusion of the corresponding main or lobar segmental bronchi to varying degrees. Central lung cancer, large airway tumors, or mediastinal tumorous lesions infiltrating or compressing the large airways, leading to stenosis, were excluded.

### 2.3 Methods

#### 2.3.1 Clinical general information

These were collected for each patient, including demographic information (gender and age), smoking history, clinical symptoms, and the final clinical diagnosis.

#### 2.3.2 High-resolution computed tomography (HRCT) of the chest

HRCT of the chest was performed using the GE LightSpeed VCT system (General Electric, USA). The imaging data were systematically analyzed to evaluate the following key features: bronchial stenosis (location), lymph node abnormalities (calcification, enlargement, and location), and the presence of additional pulmonary lesions, including exudation and consolidation, nodules, bands, interstitial changes, masses, atelectasis, pleural thickening, and pleural effusion. Lymph node abnormalities within the mediastinal window were further assessed according to the regional classification outlined in the 8th edition of the lung cancer staging system published by the International Association for the Study of Lung Cancer (IASLC) (5).

#### 2.3.3 Bronchoscopy examination

Patients are required to fast and abstain from fluids for at least six hours prior to the bronchoscopy procedure. An intravenous line is established before the operation begins. Pre-examination preparation includes the administration of sufentanil (0.2 μg/kg), propofol (1.5 mg/kg), and rocuronium (0.3 mg/kg) via intravenous injection. Once the patient’s muscles are adequately relaxed, a laryngeal mask is inserted. A T-shaped extension tube connector is then used to connect the mask to the anesthesia ventilator to ensure proper ventilation. During the procedure, additional doses of propofol (0.5–1.0 mg/kg) may be administered as needed to maintain an appropriate level of sedation. To facilitate local anesthesia, 2% lidocaine (with a total volume not exceeding 40 mL) is instilled into the trachea through the bronchoscope. Upon completion of the examination, naloxone (0.1 mg), atropine (0.5 mg), and neostigmine (1 mg) are administered intravenously to reverse the effects of the muscle relaxants and sedatives. Once the patient regains muscle strength and consciousness, the laryngeal mask is removed, and oxygen supplementation is provided. The patient is then safely transferred to the ward for continued observation. Throughout the entire procedure, continuous monitoring of the electrocardiogram, oxygen saturation, and blood pressure is essential. The BF260 electronic bronchoscope (Olympus Ltd., Tokyo, Japan) is employed to examine mucosal changes in the third to fourth levels and higher branches of the tracheobronchial tree. The locations of pigmentation, as well as areas of bronchial stenosis or obstruction, is meticulously recorded based on both the examination report and accompanying images. Whether to perform a biopsy is determined based on the findings during bronchoscopy. For a thorough examination of any identified lesions, a catheter is used to instill sterile water at room temperature directly into the lesion site. Each instillation consists of 20 mL, with a total of three instillations performed. After completing the lavage, the collected specimen volume for examination must be no less than 20 mL.

**Figure 1:**
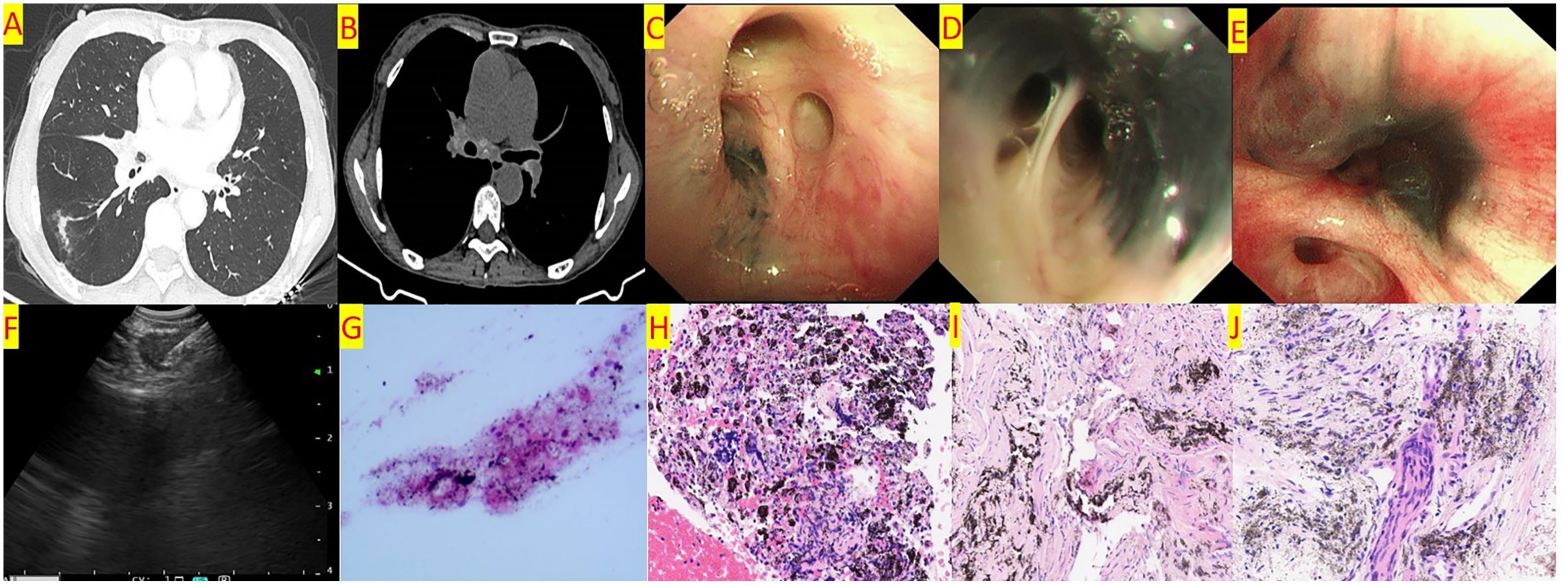
**(A)** Chest CT shows a linear high-density shadow in the right lower lobe and stenosis of the bronchial orifice of the right middle lobe. **(B)** Chest CT shows enlarged lymph nodes in the mediastinum and hilar region, with calcification visible. **(C)** Bronchoscopy shows pigmentation at the orifice of the right upper lobe anterior segment, with no stenosis of the orifice. **(D)** Bronchoscopy shows pigmentation at the orifice of the right middle lobe inner and outer segments, with mild stenosis of the orifice. **(E)** Bronchoscopy shows pigmentation at the orifice of the right lower lobe basal segment, with severe stenosis of the orifice. **(F)** EBUS-TBNA. **(G)** Cytological examination of the aspirated material from the enlarged lymph nodes shows lymphocytes and pigmentation. **(H)** Pathological examination of the aspirated material from the enlarged lymph nodes shows a background of coagulation with pigmentation, aggregation of histiocytes, and a few lymphocytes. **(I)** Histopathological examination of biopsy forceps biopsy shows widened alveolar septa, scattered lymphocyte aggregation, and pigmentation. **(J)** Histopathological examination of the enlarged lymph nodes shows focal pigmentation, and a few epithelioid cells are seen in the inflammatory necrosis. TB-DNA testing is positive.

For patients presenting with mediastinal lymph node enlargement, EBUS-TBNA (See Figure 1F) is conducted following the acquisition of informed consent. The procedure employs an advanced ultrasound bronchoscope equipped with an electronic convex array scanner (EU-M2000, Olympus Ltd., Tokyo, Japan) to examine the targeted lymph nodes. To facilitate optimal imaging, a water balloon is affixed to the ultrasound tip, operating at a scanning frequency of 7.5 MHz. The captured ultrasound images are then processed through a dedicated ultrasound image processing device (EU-C60, Olympus Ltd., Tokyo, Japan), ensuring high-resolution visualization. The puncture is performed using a 21-gauge aspiration needle (Olympus Ltd., Tokyo, Japan) under real-time ultrasound guidance. Once the needle’s entry into the target lymph node is confirmed, it is manipulated back and forth to aspirate cellular material. Prior to the puncture, the Doppler function is activated to identify and exclude vascular structures, thereby minimizing procedural risks. It is standard practice to perform three punctures on the target lymph node to ensure adequate sampling. However, if sufficient tissue samples are obtained, two punctures may suffice. The acquired histological specimens are fixed in formalin, embedded in paraffin, and sectioned for histopathological analysis.

#### 2.3.4 Evaluation of biopsy and TBNA

If malignant tumor cells or diagnostically significant cells (such as multinucleated giant cells or epithelioid cells) are identified in the pathological examination of any smear obtained from the patient’s biopsy or TBNA, the result is considered positive. Tuberculosis Deoxyribonucleic Acid (TB-DNA) testing and acid-fast staining will be do if necessary. The final diagnosis is established by integrating histopathological findings with clinical diagnostic information. Conversely, if no malignant tumor cells or diagnostically significant cells are detected across all smears during the patient’s pathological examination, the result is classified as negative (1).

#### 2.3.5 Serum T-SPOT.TB test

The T cell Spot Test for Tuberculosis Infection (T-SPOT.TB) assay was performed using a commercially available kit (Beijing Tongsheng Times Biotechnology Co., Ltd., China). Between 5 mL and 8 mL of peripheral venous blood was drawn into heparinized collection tubes (e.g., green-capped tubes). Immediately after collection, the tube was gently inverted several times to ensure thorough mixing and prevent coagulation. Samples were transported at room temperature (18–25°C) and delivered to the laboratory within 4 h of collection. Cell separation was completed within 8 h of sample collection. Freezing or refrigeration was avoided to prevent hemolysis. All procedures were conducted in a biosafety cabinet. Peripheral blood mononuclear cells (PBMCs) were isolated using density gradient centrifugation. The isolated PBMCs were resuspended in RPMI-1640 to prepare a cell suspension with a final concentration of 2.5 × 10^5^ cells/mL. Detailed steps were performed in strict accordance with the manufacturer’s instructions provided with the kit.

#### 2.3.6 Bronchoalveolar lavage fluid Xpert MTB/RIF detection

Xpert *Mycobacterium tuberculosis*/Rifampicin (Xpert MTB/RIF) assay in Bronchoalveolar Lavage Fluid (BALF) Collected BALF samples were processed by adding an equal volume of a digestion solution (4% NaOH). The mixture was vortexed for approximately 60 s until no visible clumps remained in the specimen. The assay was performed according to the instructions provided with the Xpert MTB/RIF kit (Cepheid AB, Sweden). The cartridge reaction chamber was opened, and 2 mL of the processed BALF sample was carefully added through the sample port using a sterile pipette. The loaded cartridge was then inserted into the GeneXpert system (Cepheid AB, Sweden), which automatically generated results within approximately 2 h.

### 2.4 Statistical methods

Statistical analyses were performed using SPSS 21.0 software. For measurement data conforming to a normal distribution, results were expressed as mean ± standard deviation (x̄±s), and comparisons between groups were conducted using *t*-tests. Count data were presented as case numbers and composition ratios, with *χ*^2^ tests applied for analysis. In cases where the total sample size was less than 40, Fisher’s exact test was utilized. *p* < 0.05 indicated statistical significance.

## 3 Results

### 3.1 Clinical data

Among 5,589 bronchoscopy examinations, the incidence of BAC was 1.11% (62/5,589), while that of BAF was 0.54% (30/5,589). Notably, BAF accounted for 48.4% (30/62) of all BAC cases. The BAC cohort (37 female, 25 male; mean age 67.0 ± 13.8 years, range 25–94) comprised non-BAF (*n* = 32; 17 female, 15 male; 5 smokers; mean age 67.2 ± 12.2, median 68.5, range 61.75–76.50) and BAF subgroups (*n* = 30; 20 female, 10 male; 6 smokers; mean age 67.82 ± 15.68, median 73.00, range 56.75–78.25). In contrast, the BAF group consisted of 30 cases, including 10 males and 20 females, with six smokers. The age range in this group was 56.75 to 78.25 years, with a median age of 73.00 years and an average age of 67.82 ± 15.68 years. No statistically significant differences were observed between the two groups regarding age, gender distribution (female predominance), or smoking history. However, statistically significant age-dependent increase in BAF incidence is observed. A detailed comparison of clinical symptoms between the two groups is presented in Table 1. The final clinical diagnoses for the non-BAF group were as follows: 13 cases of bacterial lung infection, 7 cases of lung tumor, 5 cases of tuberculosis, 1 case of chronic obstructive pulmonary disease, 1 case of lung abscess, 1 case of sarcoidosis, 2 cases of fungal lung infection, 1 case of *Mycobacterium avium* complex lung infection, and 1 case of pulmonary fibrosis. For the BAF group, the final clinical diagnoses included: 13 cases of bacterial lung infection, 9 cases of pulmonary tuberculosis, 5 cases of lung tumor, and 3 cases of fungal lung infection.

**Table 1:** Comparative analysis of patient symptoms and CT imaging findings between the non-BAF^#^ group and the BAF group.

Symptoms/CT imaging manifestations	Non-BAF^#^ group(32)	BAF^#^ group(30)
Symptoms		
Cough	18	14
Sputum production	7	7
Hemoptysis	5	3
Fever	4	4
Dyspnea	5	7
Lesions detected on physical examination	4	3
CT Imaging findings		
Pulmonary nodule	15	19
Pulmonary mass	11	13
Bronchial stenosis	2	10^※^
Linear or cord-like shadows	13	10
Lung consolidation	4	0^※^
Pulmonary cavity	2	0^※^
Atelectasis	2	6^※^
Bronchiectasis	5	1^※^
Pulmonary calcifications	1	7^※^
Mediastinal enlarged lymph nodes	19	27^※^
Calcified mediastinal enlarged lymph nodes	5	11^※^

※There was a statistically significant difference between the two groups. ^#^BAF, bronchial anthracofibrosis.

### 3.2 Radiological changes observed in chest CT examination

Chest CT examinations revealed several frequently observed radiological findings in both groups, including pulmonary nodules, lung masses, and linear opacities within the lungs. Notably, bronchial stenosis was more prevalent in the BAF group compared to the other cohort. Additionally, the BAF group demonstrated a higher incidence of enlarged mediastinal lymph nodes, often accompanied by calcifications. See Table 1 and Figures 1A,B.

### 3.3 Electronic bronchoscopy examination

Both cohorts exhibited right upper lobe predominance for melanin deposition. The BAF group, however, showed divergent stenosis localization, with the left lower lobe being most affected (14 cases) followed by the right upper lobe (Table 2). Further details are provided in Table 2 and Figures 1C–E.

**Table 2:** Distribution of anthracosis sites in patients from the non-BAF group or the BAF Group, and the sites of anthracofibrosis in the BAF group.

	Non-BAF^#^ group (32)	BAF^#^ group (30)	
	Sites of BAC*	Sites of BAC*	Sites of BAF
Right main bronchus	1	1	0
Right upper lobe bronchus	11	17	12
Right middle trunk bronchus	0	4	3
Right middle lobe bronchus	8	12	7
Right lower lobe bronchus	3	9	5
Left upper lobe bronchus	7	11	8
Left lower lobe bronchus	7	16	14

#BAF, bronchial anthracofibrosis; *BAC, bronchial anthracosis.

### 3.4 Results of X-PERT testing on bronchoalveolar lavage fluid

5/20 non-BAF patients and 6/15 BAF patients underwent X-PERT testing on bronchoalveolar lavage fluid (BALF) were identified as positive. All patients with positive results were ultimately diagnosed with pulmonary tuberculosis. In the BAF group, 15 patients underwent X-PERT testing on BALF, resulting in 6 positive and 9 negative findings. Similarly, all patients with positive results in this group were also confirmed to have pulmonary tuberculosis. Notably, among the 9 pulmonary tuberculosis cases in the BAF group, one case was identified as a false-negative result by X-PERT. Statistical analysis revealed no significant difference in the positivity rate of X-PERT testing between the two groups.

### 3.5 Results of the blood T-SPOT test

In the non-BAF group, 20 patients underwent T-SPOT testing, with 10 positive and 10 negative results. Among the patients ultimately diagnosed with pulmonary tuberculosis, there were 4 positive cases and 1 false-negative case. In the BAF group, 15 patients underwent T-SPOT testing, yielding 9 positive and 6 negative results. Of the positive cases, 3 were confirmed to have pulmonary tuberculosis, while no cases of pulmonary tuberculosis were identified among the negative results. Statistical analysis revealed no significant difference in the positivity rate of the T-SPOT test between the two groups.

### 3.6 Results of the pathology

In the non-BAF group, a total of 17 patients underwent bronchoscopic forceps biopsy, and 12 underwent EBUS-TBNA. In the BAF group, 15 patients underwent biopsy, and 13 underwent EBUS-TBNA. The pathological results are shown in Table 3 (See Figures 1G–J). There was no significant statistical difference in the pathological results between the two groups.

**Table 3:** Comparison of TBNA pathology and bronchoscopic biopsy pathology between the non-BAF group and the BAF group.

TBNA pathology	Non-BAF^#^ group (12)	BAF^#^ group (13)
Lymphocytes and carbon dust	9	5
Tumor cells		1
Granulomatous inflammation		3
Fungi	2	1
Lymphocytes	1	3
Biopsy Pathology	non-BAF group(17)	BAF group(15)
Interstitial fibrous tissue proliferation, pigmentation, and chronic inflammatory cell infiltration	7	4
Chronic bronchial mucosal inflammation		2
Tumor cells	7	5
Granulomatous inflammation	3	4

There was no statistically significant difference between the two groups. #BAF, bronchial anthracofibrosis.

## 4 Discussion

This retrospective study analyzed clinically confirmed cases from Henan Provincial People’s Hospital. During electronic bronchoscopy, BAC is often found, but such patients are frequently overlooked due to the presence of other comorbidities. BAF predominantly are elder, female and non-smoking patients (6, 7), with cough, sputum production, dyspnea, and hemoptysis being the most common clinical symptoms (6, 8). The statistical description of symptoms in this study is consistent with the literature, but when it comes to individual patients, these symptoms do not have significant differential diagnostic significance from other respiratory diseases. The literature suggests that chest CT of BAF patients shows non-continuous segmental bronchial stenosis, with the right middle lobe being the most susceptible, often accompanied by mediastinal, hilar, and interlobar lymph node enlargement and calcification (9). However, in this study, among the 30 BAF group patients, only 10 showed stenosis on chest CT, and 2 patients in the BAC group also showed bronchial stenosis. Subsequent bronchoscopy revealed 49 sites of bronchial stenosis in the BAF group, with the most common sites being the left lower lobe and right upper lobe which is inconsistent with the results of other domestic authors (10). Although bronchoscopy is more reliable for the diagnosis of BAF, chest CT can indicate the enlargement of mediastinal lymph nodes. Our study revealed significantly more frequent mediastinal lymphadenopathy in BAF versus BAC (27/30 vs. 19/62 cases) and greater lymph node calcification (11 vs. 5 cases). It is evident that chest CT examination can indicate bronchial stenosis, enlargement or calcification of mediastinal or hilar lymph nodes, and abnormalities of lung parenchyma. Additionally, it is convenient, fast, and non-invasive, making it an important supplementary examination method to bronchoscopy.

There are various causes of bronchial stenosis accompanied by distal atelectasis and mediastinal lymph node enlargement, and BAF is one of them. Since patients with BAF may also have other diseases, it is necessary to perform bronchoscopy or TBNA for patients suspected of having other diseases on chest CT. Bronchoscopy can directly observe the location of bronchial mucosal anthracosis, the position of bronchial stenosis or obstruction, and other pathological conditions, which is intuitive for the diagnosis of BAF. At the same time, through bronchoscopy, biopsies and/or bronchoalveolar lavage can be performed on the lesion areas to obtain histological specimens for pathological examination, which helps to clarify the diagnosis of the lesion. In addition to the characteristic changes of airway mucosal interstitial fibrous tissue proliferation, pigmentation, and chronic inflammatory cell aggregation seen in the biopsy pathology of both BAC and BAF (6, 11), some patients also found tumor cells or granulomatous lesions. However, for BAF patients, due to the presence of bronchial stenosis or obstruction, the bronchoscope may not be able to pass through or obtain meaningful lavage fluid from the required lesion site, which can explain the negative X-PERT test results in the lavage fluid of BAF patients with tuberculosis. Additionally, the bronchoscopy biopsy pathology showed interstitial fibrous tissue proliferation, pigmentation and chronic inflammatory cell infiltration for 7 non-BAF patients compared with 4 BAF patients.2 cases were chronic bronchial mucosal inflammation, which may be because the non-BAF group did not have bronchial stenosis and was more likely to obtain relevant tissues. For patients with bronchial stenosis or mediastinal lymph node enlargement, TBNA can be used to obtain pathology. Performing puncture under real-time ultrasound guidance can improve the accuracy of diagnosis and provide a basis for formulating personalized treatment plans. In this observation, the pathology obtained by EBUS-TBNA in both groups was mostly lymphocytes and carbon dust (9 cases in the non-BAF group and 5 cases in the BAF group); the BAF group also had tumors (1 case) and granulomas (3 cases), which provided evidence for disease diagnosis. Compared with traditional mediastinoscopy, EBUS-TBNA has the advantages of less trauma and fewer complications. It does not require thoracotomy, reducing the patient’s suffering and postoperative recovery time, and is especially suitable for elderly patients or those with severe comorbidities.

There are various theories regarding the pathogenesis of BAF. Some attributes it to tuberculosis infection, while another links it to chronic inhalation of household smoke and dust, leading to phagocytosis by macrophages. However, the exact pathogenesis remains unclear. Most researchers agree that BAF is closely related to tuberculosis and endobronchial tuberculosis. It is thought that the bronchial stenosis in this condition may be secondary to an excessive immune response of the body to lymphatic or adjacent pulmonary tuberculosis antigens, or it may originate from intraluminal infection due to reflux of bacteria within the bronchial lumen, or it could be caused by external compression from enlarged mediastinal lymph nodes (1, 12, 13). In terms of treatment, Chung et al. believe that anti-tuberculosis treatment should be administered regardless of whether *Mycobacterium tuberculosis* is detected (1). There are case reports of young patients with BAF and pulmonary tuberculosis showing recovery of the narrowed segments after anti-tuberculosis treatment (14). In this study, among the confirmed cases of pulmonary tuberculosis, there were 5 cases (5/32) in the non-BAF group and 9 cases (9/30) in the BAF group, suggesting that BAF may have a higher incidence of tuberculosis; and from the overall pathology, the BAF group showed a relatively higher frequency of granulomatous inflammation when combining biopsy and TBNA results. The results of bronchoalveolar lavage fluid X-PERT and blood T-spot tests indicate that these tests have certain value in diagnosing pulmonary tuberculosis. Some researchers also believe that some BAF patients do not have active or chronic tuberculosis but are associated with Chronic Obstructive Pulmonary Disease, pneumonia, or malignant tumors, and other factors such as smoking and air pollution may also be related to this disease (15–18). In this study, the final confirmed cases encompassed not only tuberculosis but also other pulmonary infectious lesions, as well as fungal infections and tumor cases in varying proportions. As demonstrated in our findings, these testing methods are not entirely accurate. None of the diagnostic tests proved fully reliable, and factors like airway narrowing in BAF occasionally produced false-negative results. Consequently, in clinical diagnosis, it is imperative not to rely solely on a single testing method. Rather, a comprehensive evaluation should integrate the patient’s clinical symptoms, chest CT findings, additional laboratory tests, and histopathological examinations to enhance diagnostic precision.

This study undertook a preliminary investigation into the incidence rates of Bronchial Anthracotic Changes (BAC) and Bronchial Anthracofibrosis (BAF) in bronchoscopy examinations conducted in Henan in China. The findings revealed incidence rates of 1.11% for BAC and 0.54% for BAF, with BAF accounting for 48.4% of patients diagnosed with anthracosis. These figures are notably lower than the reported incidence range of BAF, which varies between 2 and 20% in existing literature (7). In contrast, a large-scale study conducted in Iran involving 14,300 individuals identified 487 cases of anthracosis (3.4%), among which 291 were classified as BAF patients (2.0%) (7). Given that this Iranian study was a large-sample, multicenter, cross-provincial investigation with a relatively balanced age distribution, its reported incidence of anthracosis differs from our findings. However, the proportion of BAF among anthracosis patients in the Iranian study (59.8%) closely aligns with our results, suggesting a potentially consistent relationship between anthracosis and BAF across different populations. The etiology of BAF is thought to be multifactorial, potentially linked to exposures such as biomass fuel combustion, coal smoke inhalation, and tuberculosis infection (1, 13, 19). Although the present study lacks detailed occupational and exposure-related data, it is noteworthy that 74.2% of BAC patients were elderly individuals aged over 60 years. Furthermore, all patients included in the analysis were residents of Henan in China, where coal and biomass were common household fuels 30 to 40 years ago. It implies particularly women, may have had significant historical exposure to biomass fuels or coal smoke (13, 20). Overall, this study provides valuable insights into the epidemiological patterns of BAC and BAF in Henan, China, and highlight the potential role of environmental and lifestyle factors in their pathogenesis. Further research incorporating detailed exposure histories and longitudinal data will be essential to deepen our understanding of these conditions.

BAF is a clinical syndrome characterized by distinct clinical, radiological, and pathological features. This study further highlights the diagnostic value of EBUS-TBNA in identifying BAC/BAF associated with mediastinal enlarged lymph node lesions. Analysis of combined BAC and BAF cohorts suggests that BAF can coexist not only with active tuberculosis but also with other pulmonary infections or malignancies. Additionally, other factors such as smoking and air pollution may further contribute to the development or progression of the condition.

## Data Availability

The raw data supporting the conclusions of this article will be made available by the authors, without undue reservation.
